# Application of a ω-3 Desaturase with an Arachidonic Acid Preference to Eicosapentaenoic Acid Production in *Mortierella alpina*

**DOI:** 10.3389/fbioe.2017.00089

**Published:** 2018-01-22

**Authors:** Chengfeng Ge, Haiqin Chen, Tiantian Mei, Xin Tang, Lulu Chang, Zhennan Gu, Hao Zhang, Wei Chen, Yong Q. Chen

**Affiliations:** ^1^State Key Laboratory of Food Science and Technology, Jiangnan University, Wuxi, China; ^2^School of Food Science and Technology, Jiangnan University, Wuxi, China; ^3^National Engineering Research Center for Functional Food, Jiangnan University, Wuxi, China; ^4^Beijing Innovation Centre of Food Nutrition and Human Health, Beijing Technology and Business University (BTBU), Beijing, China; ^5^Department of Cancer Biology, Wake Forest School of Medicine, Winston-Salem, NC, United States

**Keywords:** ω-3 fatty acid desaturase, eicosapentaenoic acid, *Mortierella alpina*, transformation, culture variables

## Abstract

In the industrial oleaginous fungus *Mortierella alpin*a, the arachidonic acid (AA; C20:4; ω-6) fraction can reach 50% of the total fatty acids (TFAs) *in vivo*. However, the eicosapentaenoic acid (EPA; C20:5; ω-3) fraction is less than 3% when this fungus is cultivated at a low temperature (12°C). Omega-3 fatty acid desaturase is a key enzyme in ω-3 long-chain polyunsaturated fatty acids biosynthesis pathways. To enhance EPA production, we transformed the ω-3 fatty acid desaturase (PaD17), which exhibits strong Δ-17 desaturase activity, into *M. alpina*, thus increasing the AA to EPA conversion rate to 49.8%. This PaD17-harboring *M. alpina* reconstruction strain produced 617 mg L^−1^ of EPA at room temperature in broth medium, this yield was increased to 1.73 g L^−1^ after culture medium optimization (i.e., about threefold higher than that under original culture conditions), with concomitant respective increases in dry cell weight and TFA content to 16.55 and 6.46 g L^−1^. These findings suggest a new platform for the future industrial production of EPA.

## Introduction

Long-chain polyunsaturated fatty acids (LC-PUFAs), particularly the ω-3 LC-PUFAs eicosapentanoic acid (EPA) and docosahexanoic acid (DHA), are essential nutrients for humans (Das and FAMS, 2003; Swanson et al., 2012). Numerous clinical studies have documented the wide-ranging health benefits conferred by ω-3 LC-PUFAs against various symptoms and diseases, including asthma, depression, diabetes, immune disorders, cardiovascular disease, and cancer (Hirahashi et al., 2014; Jump et al., 2015; Maehre et al., 2015; Miyata and Arita, 2015). Accordingly, the demand for ω-3 LC-PUFAs has increased rapidly in the pharmaceutical, medical, and nutritional sectors. EPA and DHA cannot be synthesized *de novo* in mammals, but must be obtained either directly through dietary sources or indirectly through the further desaturation and elongation of other PUFAs widely available in the diet, such as linoleic acid (LA) or α-linolenic acid (ALA). In contrast, microorganisms and phytoplankton, such as diatoms (Hamilton et al., 2014), various types of algae, cyanobacteria, oomycetes, and fungi can synthesize LC-PUFAs *de novo* (Xue et al., 2013a; Vadivelan and Venkateswaran, 2014; Xie et al., 2015). The expanding global population has increased the pressure on ocean fishery resources to supply foods enriched in ω-3 fatty acids (Baik et al., 2010), leading to the recognition of current wild fish harvesting and commercial fish farming practices are not sustainable over the long term. Furthermore, pollution remains a concern regarding fish-derived ω-3 fatty acid oils (Jacobs et al., 1998). Therefore, a sustainable supply of clean EPA and/or DHA could reduce the pressure on ocean resources and protect the integrity of ω-3 fatty acid products and the environment.

**Figure 1:**
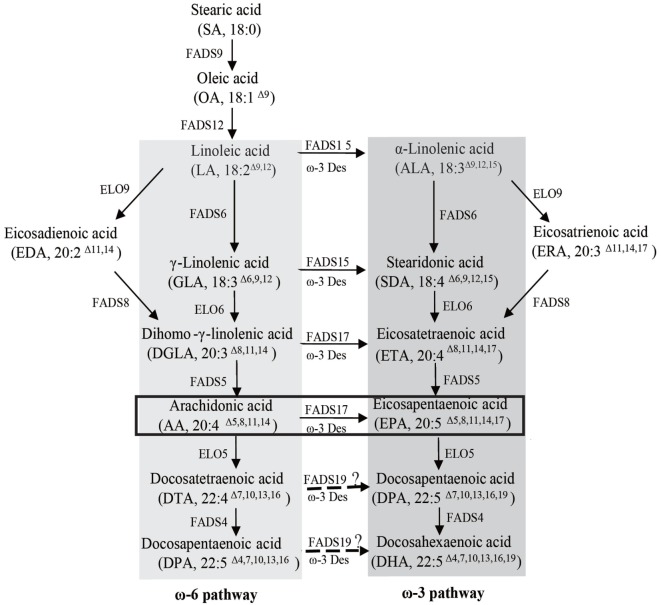
Fatty acid synthesis in *Mortierella alpina*. The ω-6 and ω-3 pathways are highlighted by dark gray and light gray boxes, respectively. The ω-3 desaturase *oPaFADS17* plays a major role in the pathway highlighted by the black box. FADS9, delta 9 desaturase; ELO9, delta 9 elongase; FADS12, delta 12 desaturase; FADS6, delta 6 desaturase; FADS15, delta 15 desaturase; ELO6, delta 6 elongase; FADS8, delta 8 desaturase; FADS5, delta 5 desaturase; FADS17, delta 17 desaturase; ELO5, delta 5 elongase; FADS19, delta 19 desaturase; FADS4, delta 4 desaturase; ELO7, delta 7 elongase.

Various organisms, including plants (Adarme-Vega et al., 2014; Ruiz-Lopez et al., 2015), algae, and fungi, are currently under investigation as potential hosts for sustainable commercial EPA and DHA production. Recent studies have reported the reconstitution of the ω-3 LC-PUFAs biosynthetic pathway in yeast, and have engineered the oleaginous yeast strain *Yarrowia lipolytica* for commercial EPA production (Nisha et al., 2009; Sakuradani, 2010). The *Y. lipolytica* can produce EPA levels equivalent to 56.6% of total fatty acid (TFA) and accumulate lipid content as high as 30% of the dry cell weight after 6 days of culture in high-glucose medium. The oleaginous filamentous fungus *Mortierella alpina*, which can accumulate a fatty acid content of up to 50% of the dry cell weight (Sakuradani et al., 2013), has been used safely for the industrial production of arachidonic acid (AA) for use in infant formulas (Streekstra, 1997). *M. alpina* has been recognized as a potential source for the commercial production of EPA, which can be synthesized from AA *via* ω-3 fatty acid desaturase-catalyzed dehydrogenation (Figure 1). Ando et al. (2009) overexpressed the endogenous ω-3 fatty acid desaturase gene in *M. alpina* 1S-4 and demonstrated an improved EPA yield, with a maximum level of 40% of TFA after cultivation at 12°C for 16 days.

Considerable efforts have been made to identify and characterize ω-3 desaturases from various sources. Early studies found that plant ω-3 desaturases exclusively desaturate 18-carbon ω-6 PUFAs (Venegas-Caleron et al., 2006; Niu et al., 2008) and that a *Caenorhabditis elegans* ω-3 desaturase preferentially desaturated 18-carbon PUFAs vs. 20-carbon substrates (Meesapyodsuk et al., 2000). Several types of ω-3 desaturases with high Δ-17 desaturase activity were recently discovered in successive studies. For example, Pereira et al. (2004) cloned a novel ω-3 desaturase (SDD17) from *Saprolegnia diclina*, and expression of *sdd17* gene in yeast revealed that the encoded protein exclusively desaturated 20-carbon PUFAs, with a preference for AA. Another newly discovered ω-3 desaturase (OPIN17) from *Phytophthora infestans*, converted 30.94% of AA into EPA (Fu et al., 2013). Recently, Xue et al. (2013b) identified three ω-3 desaturases from *Pythium aphanidermatum* (PaD17), *Phytophthora sojae* (PsD17), and *Phytophthora ramorum* (PrD17). Of these three enzymes, PaD17 exhibited strong Δ-17 desaturase activity and the highest AA to EPA conversion rate, yielding a 56.6% EPA concentration among TFA in genetically engineered *Y. lipolytica*.

Large-scale fermentation-based EPA production requires the use of an inexpensive medium to reduce costs. As reported, the lipid contents and fatty acid profiles of *M. alpina* are strongly affected by environmental conditions (Nisha and Venkateswaran, 2011). We first expressed PaD17 in *Saccharomyces cerevisiae* to verify its substrate preference and subsequently transformed the *pad17* gene into *M. alpina*, leading to the accumulation of an EPA level of approximately 18% of TFA at room temperature. In order to increase EPA yields and decrease fermentation costs, we then optimized culture variables through single-factor experimentation and an orthogonal experiment. This report describes our attempts to formulate suitable media for mycelial biomass, lipid, and EPA production by *M. alpina* recombinant and represents a crucial step for EPA industrial production.

## Materials and Methods

### Strains, Media, and Growth Conditions

The wild-type *M. alpina* ATCC 32222 strain was purchased from American Type Culture Collection (ATCC, Manassas, VA, USA) and conserved in our lab; the *M. alpina* uracil-auxotrophic strain (CCFM501, Culture Collection of Food Microorganisms, Jiangnan University, Jiangsu Province, PR China) was modified from wild-type *M. alpina* in our lab (Hao et al., 2014). *Agrobacterium tumefaciens C58C1* was provided by Yasuyuki Kubo (Kyoto Prefectural University, Kyoto, Japan) and used as a transfer DNA (T-DNA) donor for fungal transformation. The *Escherichia coli* TOP10 strain was used for plasmid storage. The pYES2/NT C plasmid containing the galactose-inducible GAL1 promoter was purchased from Invitrogen (Carlsbad, CA, USA); the pBIG2-ura5s-ITs plasmid was modified from pBIG2RHPH2 (provided by Yasuyuki Kubo).

*Saccharomyces cerevisiae* cultures were grown in yeast extract/peptone/dextrose medium at room temperature for 2 days with constant agitation at 200 rpm. The *S. cerevisiae* strain INVSc 1 (his3Δ1/his3Δ1 leu2/leu2 trp1-289/trp1-289 ura3-52/ura3-52) was grown on YEP medium (10 g L^−1^ tryptone, 10 g L^−1^ yeast extract, and 5 g L^−1^ NaCl) at 28°C. Wild-type *M. alpina* ATCC 32222 was grown on GY solid medium (30 g L^−1^ glucose, 5 g L^−1^ yeast extract, 2 g L^−1^ KNO_3_, 1 g L^−1^ NaH_2_PO_4_, and 0.3 g L^−1^ MgSO_4_⋅7H_2_O). The auxotrophic CCFM 501 strain was maintained on GYU solid medium (30 g L^−1^ glucose, 5 g L^−1^ yeast extract, 2 g L^−1^ KNO_3_, 1 g L^−1^ NaH_2_PO_4_, and 0.3 g L^−1^ MgSO_4_⋅7H_2_O supplemented with 0.05 g L^−1^ uracil) at 25°C. Transformation media included synthetic complete (SC) medium, minimal medium (MM), and induction medium (IM) (Michielse et al., 2008). For fatty acid accumulation, each transformant and wild-type *M. alpina* were grown for 7 days at 28°C in broth medium (50 g L^−1^ glucose, 5 g L^−1^ yeast extract, 10 g L^−1^ KNO_3_, 1 g L^−1^ KH_2_PO_4_, and 0.25 g L^−1^ MgSO_4_⋅7H_2_O, pH 6.0).

### Yeast Strain Construction

The PaD17 sequence was optimized based on the codon preferences of *S. cerevisiae*; the resulting codon-optimized gene, *oPAD17* (1,077 bp in length), was synthesized and sub-cloned into the pUC57-simple vector. The *oPAD17* F/*oPAD17* R primer pair (Table 1) was used to amplify *oPAD17* under PCR cycling conditions of 94°C for 3 min, 30 cycles of amplification at 94°C for 30 s, 58°C for 30 s, and 68°C for 1.5 min, and a final extension at 68°C for 7 min. The *oPAD17* coding sequence fanked with restriction endonuclease sites *EcoR* I and *Xho* I was ligated into pYES2/NT C (with T7 promoter and T7 terminator) to yield the plasmid pYES2/NT C-*oPAD17*. The insert was confirmed by sequencing, after which the plasmid containing *oPAD17* was electro-transformed into *E. coli* TOP10 for storage. The haploid strain INVSc1 was transformed with pYES2/NT C-*oPAD17* and selected on minimal uracil-free agar plate to yield the strain INVSc 1 (pYES2/NT C-*oPAD17*).

**Table 1:** Primers used in this study.

Primer name	Restriction enzyme	Oligonucleotide sequence (5′–3′)	Function
***Saccharomyces cerevisiae***			
*oPAD17-*F	*EcoR* I	CATGTAGAATTCATGGCTTCGTCCACCGTTG	*oPAD17* amplification for plasmid construction
*oPAD17-*R	*Xho* I	TTACGACTCGAGTTAGTTAGCCTTGGTCTTGGCAG	
q-*oPAD17-*F	–	CTTCGTCCACCGTTGCTG	qPCR for *oPAD17* transcription level measurement
q-*oPAD17-*R	–	AGCCAGCGATTCCGAGA	
18S F	–	AATCATCAAAGAGTCCGAAGACATTG	The internal control gene for qPCR
18S R	–	CCTTTACTACATGGTATAACTGTGG	

***Mortierella alpine***			
*oPaFADS17-*F	*Hind* III	ATACCCAAGCTTCAATGGCTTCGTCCACCGTTG	*oPaFADS17* amplification for plasmid construction
*oPaFADS17*-R	*Xho* I	AGCGTCCTCGAGTTAGTTAGCCTTGGTCTTGGCAG	
q-*oPaFADS17*-F	–	CTTCGTCCACCGTTGCTG	qPCR for *oPaFADS17* transcription level measurement
q-*oPaFADS17-*R	–	AGCCAGCGATTCCGAGA	
18SRTF	–	CGTACTACCGATTGAATGGCTTAG	The internal control gene for qPCR
18SRTR	–	CCTACGGAAACCTTGTTACGACT	
HisproF	–	CACACACAAACCTCTCTCCCACT	T-DNA insert detection for binary plasmid construction
TrpCR	–	CAAATGAACGTATCTTATCGAGATCC	

### Transcription Level Analysis of PaD17 Gene in *S. cerevisiae* Transformants

Total RNA was isolated from pelleted transformants using TRIzol reagent (Invitrogen) and reverse-transcribed using the Prime Script RT reagent kit (Takara, Otsu, Shiga, Japan) according to the manufacturers’ instructions. The q-*oPAD17* F/q-*oPAD17* R primer pair used for RT-qPCR is summarized in Table 1. An ABI-Prism 7900 sequence detection system and Power SYBR Green PCR Master Mix (both Applied Biosystems, Foster City, CA, USA) were used for the RT-qPCR analysis according to the manufacturer’s instructions. The reaction mixtures comprised 10 µL of SYBR Green PCR Master Mix, 0.5 µL of each primer pair, 8 µL of distilled water, and 1 µL of DNA template or distilled water for a no-template control. The PCR cycling conditions were 50°C for 2 min, 95°C for 10 min, and 40 cycles of amplification at 95°C for 15 s and 60°C for 30 s. The housekeeping gene 18S rRNA was used as the normalization standard for gene expression. The primer pair 18S F/18S R was used as indicated in Table 1.

### Western Blot Analysis of *S. cerevisiae* Transformants

Western blot analysis was performed as described previously (Chen et al., 2013). One hundred micrograms of cellular protein extract per lane (in duplicate) were subjected to SDS-PAGE (12% gel), followed by western blotting analysis or coomassie blue staining. PVDF membranes (Millipore, Billerica, MA, USA) were used for western blot analysis. To detect recombinant proteins, membranes were incubated with an anti-His primary antibody (Nanjing GenScript Biotechnology Corporation, Nanjing, PR China), followed by a horseradish peroxidase-conjugated goat anti-mouse IgG (Nanjing GenScript Biotechnology Corporation, Nanjing, PR China).

### ω-6 Fatty Acid Substrate Supplementation

INVSc 1 (pYES2/NT C-*oPAD17*) strains were grown on SC medium. Overnight cultures were diluted to an optical density at 600 nm (OD_600_) of 0.4 and subsequently aliquoted into three 20-ml cultures containing 0.2 mM each of LA, GLA, DGLA, and ARA, respectively. Next, different concentrations of AA (0.05, 0.1, and 0.2 mM) were added to the culture media to investigate the AA to EPA conversion activity. After incubation for another 48 h, the cultures were harvested by centrifugation and subjected to further analysis.

### T-DNA Binary Vector Construction

The PaD17 gene was codon-optimized based on the codon preferences of *M. alpina*; the resulting gene, *oPaFADS17* (1,077 bp in length, GenBank accession No. KT372000) was synthesized and sub-cloned into the pUC57-simple vector. The *oPaFADS17* F/*oPaFADS17* R primer pair (Table 1) was used to amplify *oPaFADS17* under PCR conditions of 94°C for 3 min, 30 amplification cycles of 94°C for 30 s, 58°C for 30 s, and 68°C for 1.5 min, and a final extension step at 68°C for 7 min. The T-DNA binary vector pBIG2-ura5s-ITs, which contains a ura5s cassette as the selection marker, contains the His550 promoter and trpCt transcription terminator, was used as the backbone for expression vector construction, The *oPaFAD17* coding sequence fanked with restriction endonuclease sites *Hind* III and *Xho* I was cloned into this vector to generate pBIG2-ura5s-*oPaFADS17*. Validated binary vectors carrying the *oPaFADS17* gene were electrotransformed into *E. coli* TOP10 and *A. tumefaciens* C58C1 for storage or *A. tumefaciens*-mediated transformation (see below).

### *A. tumefaciens*-Mediated Transformation

*Agrobacterium tumefaciens*-mediated transformation was performed using an appropriately modified version of a previously described protocol (Ando et al., 2009). Uracil-auxotrophic *M. alpina* CCFM501 spores were harvested from 30-day cultures grown on GY agar supplemented with 0.05 g L^−1^ uracil and suspended. Positive *A. tumefaciens* transformants carrying pBIG2-ura5s-*oPaFADS17* were confirmed by PCR with the HisproF/TrpCR primer pair (Table 1). *A. tumefaciens* transformants were grown at 28°C for 48 h to an OD_600_ of 1.2 in MM medium supplemented with 100 µg mL^−1^ kanamycin and 100 µg mL^−1^ rifampicin.

Bacterial cells were harvested by centrifugation, washed with fresh IM medium, and diluted to an OD_600_ of 0.3 in 20 ml of fresh IM. After preincubation for 12 h at 25°C with shaking (200 rpm), the culture was diluted to an OD_600_ of 0.4–1.2, after which 100 µL of bacterial cell suspension was mixed with an equal volume of a spore suspension (10^7^/100 μL) and spread on cellophane membranes. The membranes were placed on cocultivation medium (similar to IM, except with a glucose concentration of 0.9 g L^−1^) and incubated at 23°C for approximately 36 h in a dark incubator. After cocultivation, the membranes were transferred to uracil-free SC agar plates containing 100 µg mL^−1^ cefotaxime and 100 µg mL^−1^ spectinomycin and cultured at 18°C for 12 h to inhibit the *A. tumefaciens* growth, and then at 25°C until colonies appeared. Hyphae from visible fungal colonies were then transferred to fresh uracil-free SC agar plates. This transfer process was repeated three times to obtain stable transformants. All experiments were performed in triplicate.

### Preparation of Genomic DNA of *M. alpina* Transformants

Transformants were cultivated in liquid broth medium at 28°C with shaking at 200 rpm for 7 days. *M. alpina* mycelia were harvested by filtration and washed twice with sterile water. The integration of T-DNA into *M. alpina* genomic DNA was verified by PCR. One pair of primers (HisproF1/TrpCR1; Table 1) was designed to target promoter and terminator-specific regions and confirm successful gene integration. The PCR conditions were 94°C for 3 min, 30 amplification cycles of 94°C for 30 s, 55°C for 30 s, and 72°C for 2 min, and a final extension step of 72°C for 10 min. Positive transformants were expected to yield 818 and 1,244 bp fragments.

### PaD17 Gene Transcript-Level Analysis in *M. alpina* Transformants

*Mortierella alpina* strains were cultivated in broth medium at 28°C with shaking at 200 rpm for 7 days. *M. alpina* mycelia were harvested by filtration and immediately frozen in liquid nitrogen for storage. TRIzol-mediated total RNA extraction and RT-qPCR were performed as described above. The primer pairs used for RT-qPCR were shown in Table 1. The internal control gene 18S rRNA was used as the normalization standard for gene expression.

### Effect of Culture Media to EPA Production

Single-factor experiments and a designed orthogonal experiment were performed to increase the EPA yield. Part 1 involved an investigation of the effects of different carbon sources, including glucose, corn starch, soluble starch, potato starch, and glycerol, on dry biomass, lipid, and EPA production. Part 2 compared an inexpensive defatted soybean meal (DSMO) with a costly yeast extract as organic nitrogen sources. Part 3 evaluated various concentration of inorganic nitrogen KNO_3_. Finally, the effects of EPA production were investigated through an orthogonal experimental design in which the following three factors were analyzed: carbon source (factor A), DSOM concentration (factor B), and KNO_3_ concentration (factor C). An OA_9_ (3^3^), or orthogonal array of three factors and three levels, was employed to assign the factors for consideration, as shown in Table 2. Nine trials were performed to complete the optimization process according to the OA_9_. A range analysis was used to determine the optimal EPA production conditions.

**Table 2:** Levels and factors affecting EPA production.

Level	Factors		
	Carbon source A (50 g L^**−**1^)	DSOM concentration B (g L^**−**1^)	KNO_3_ concentration C (g L^**−**1^)
1	Glucose	30	0
2	Corn starch	50	5
3	Glycerol	70	10

*DSOM, defatted soybean meal, used as organic nitrogen*.

### Range Analysis

A range analysis includes two parameters, *K_ji_* and *R_j_*. *K_ji_* is defined as the sum of the indexes of all levels in each factor *j* and *k_ji_* is the average value of each experimental factor at the same lever *i*; *R_j_* is defined as the range between the maximum and minimum values of *k_ji_*. As *k_ji_* is used to evaluate the importance of factors, a large *R_j_* indicates greater importance of a particular factor.

### Fatty Acid Methyl Ester (FAME) Analysis

Fatty acids were extracted and methyl-esterified from approximately 50 mg of freeze-dried cells, as described previously (Chen et al., 2013). FAME profiles were analyzed using gas chromatography/mass spectrometry (GC/MS; GC-2010 Plus; GCMS-QP2010 Ultra, Shimadzu Co., Kyoto, Japan) with a 30 m × 0.25 mm Rtx-Waxetr column (film thickness: 0.25 µm) and helium as the carrier gas. The following temperature programme was set: 40°C for 5 min, ramp to 120°C at 20°C per min, ramp to 190°C at 5°C per min, hold for 5 min, ramp to 220°C at 5°C per min, and hold for 17 min. Fatty acid quantification was performed using peak-height area integrals. Pentadecanoic acid was used as the internal standard to quantify FAMEs with aliphatic chains ≤18, and nonadecanoic acid was used as the internal standard to quantify FAMEs with aliphatic chains >18.

### Statistical Analysis

All experiments were performed independently at least three times, and the mean values ± SDs were presented. Statistical analysis was performed with one-way analysis of variance with Tukey’s test with SPSS 20, and *P* < 0.05 was considered to indicate statistical significance.

## Results

### Transcription and Translation of PaD17 in *S. cerevisiae*

**Figure 2:**
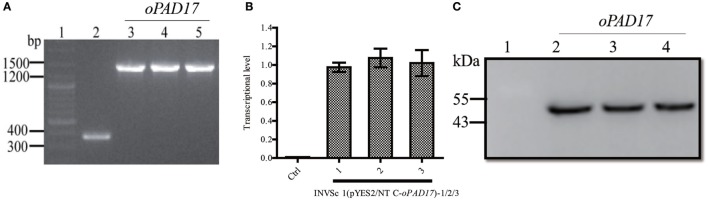
Identification of transformants by PCR and analysis of transcription and translation of PaD17 in INVSc 1 (pYES2/NT C-*oPAD17*-1/2/3). **(A)** Map of fragment bands generated by PCR, 1: nucleic acid marker, 2: INVSc1-pYES2/NT C control strains (368 bp), 3–5: INVSc 1 (pYES2/NT C-*oPAD17*-1/2/3) (1,448, 1,080 + 368 bp); **(B)** transcription levels of PaD17; **(C)** translation levels of PaD17, 1: NVSc1-pYES2/NT C control strains, 2–4: INVSc 1 (pYES2/NT C-*oPAD17*-1/2/3) (45 kDa).

The optimized *oPAD17* gene was used to construct pYES2/NT C-*oPAD17*, which was successfully transformed into *S. cerevisiae* (Figure 2A). RT-qPCR (Figure 2B) and western blotting (Figure 2C) revealed normal levels of PaD17 transcription and translation, indicating successful target gene transcription and expression in the recombinant strain.

### Substrate Preference of PaD17

**Figure 3:**
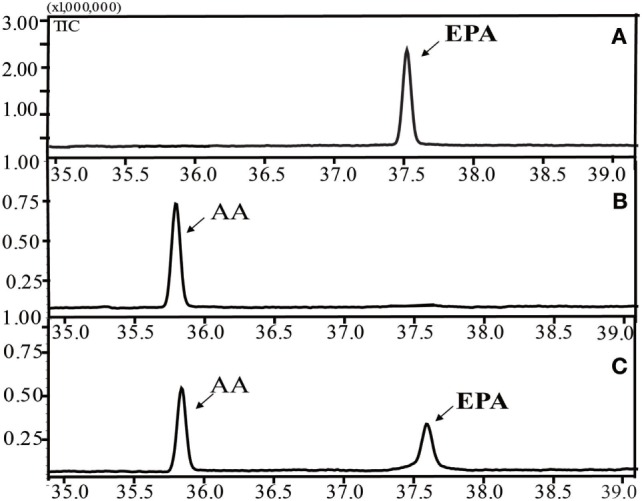
Gas chromatogram of fatty acid methyl esters from the lipid fractions of the yeast transformant INVSc 1 (pYES2/NT C-*oPAD17*). **(A)** EPA standard; **(B)** INVSc1-pYES2/NT C control strain; **(C)** INVSc 1(pYES2/NT C-*oPAD17*) strain.

Figure 3 shows the successful functional expression of INVSc 1 (pYES2/NT C-*oPAD17*), along with representative GC-MS analyses of FAMEs in yeast cultures. Gas chromatograms (Figure 3C) detected a peak corresponding to the C20:5 (5,8,11,14,17) standard (Figure 3A) that was not present in the INVSc1-pYES2/NT C control strain (Figure 3B). To gain qualitative insight into the substrate requirements of the *oPAD17*-encoded protein, various possible fatty acid substrates were supplied to yeast cultures expressing this enzyme.

**Figure 4:**
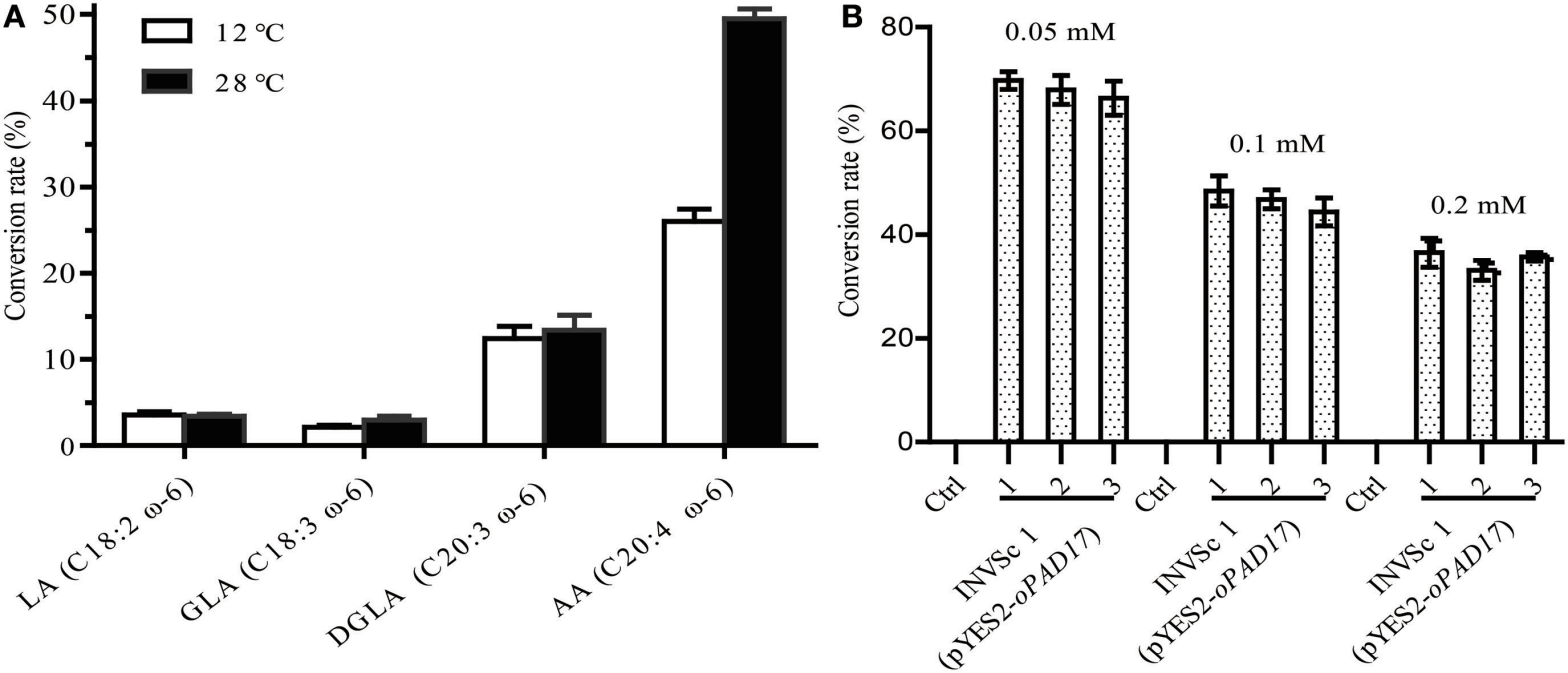
The conversion rate of INVSc 1 (pYES2/NT C-*oPAD17*) with exogenous fatty acids supplementation. **(A)** Is for different fatty acids at different temperatures; **(B)** is for different arachidonic acid concentration. Conversion rate was calculated as 100% × Product/(Product + Substrate).

INVSc 1 (pYES2/NT C-*oPAD17*) strains were grown at room temperature (28°C) on YEP medium supplemented with linoleic acid (LA, C18:2, ω-6), γ-linolenic acid (GLA, C18:3, ω-6), dihomo-γ-linolenic acid (DGLA, C20:3, ω-6), and AA (C20:4, ω-6) (Figure 4A). LA, GLA, DGLA, and AA were converted into ALA (C18:3, ω-3), stearidonic acid (SDA, C18:4, ω-3), eicosateteraenoic acid (ETA, C20:4, ω-3) and eicosapentaenoic acid (EPA, C20:5, ω-3), respectively. Although the *oPAD17* product exhibited poor 18 C-PUFA conversion efficiency (<5%), it exhibited much better efficiency for 20 C-PUFAs (>10%), especially AA for which the conversion rate was almost 50%. We also determined desaturase activities at a low temperature (12°C), and observed lower conversation rate (Figure 4A) relative to those obtained at room temperature. Furthermore, we evaluated the effects of different AA concentration on desaturase activity (Figure 4B). As shown in Figure 4B, the AA to EPA conversion rate decreased with increasing AA concentration. When exposed to 0.05 mM AA, INVSc 1 (pYES2/NT C-*oPAD17*) strains exhibited conversion rate as high as 68%, consistent with the reported rate for *Y. lipolytica*.

### Transformation of *oPaFADS17* into *M. alpina*

The optimized *oPaFADS17 g*ene was heterologously expressed in the *M. alpina* auxotrophic CCFM 501 strain, and putative transformants were selected randomly by subculture on uracil-free SC medium. Stable transformants were then obtained by successive sub-culturing on uracil-free SC agar plates for three generations, and were transferred to GY agar plates for further analysis. We eventually obtained six mitotically stable *oPaFADS17* transformants.

**Figure 5:**
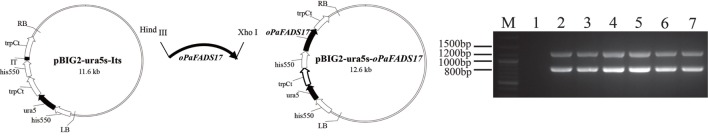
Construction of the binary vector pBIG2-ura5s-*oPaFADS17*. The his 550 promoter fragment was derived from *Mortierella alpina* ATCC 32222. The trpCt transcription terminator region was derived from *Aspergillus nidulans*. LB, left border; RB, right border. M, marker; lane 1, *M. alpina* (negative control); lanes 2–7: *M. alpina*-*oPaFADS17*-(1–6). Bands at 818 and 1,244 bp indicate the presence of the *oPaFADS17* gene.

The T-DNA region of the binary vector pBIG2-ura5s-*oPaFADS17* (Figure 5) should have integrated into the genomic DNA of *M. alpina* during the *A. tumefaciens* transformation process. Accordingly, we verified the presence of the ura5 and *oPaFADS17* expression cassettes in the transformant genomes using PCR. The presence of two fragments (818 and 1,244 bp) confirmed that the *oPaFADS17* expression cassettes were randomly inserted into the *M. alpina* genome, whereas the control strain (wild-type *M. alpina*) did not yield corresponding DNA fragments (Figure 5).

### Transcript Levels and Fatty Acid Analysis of MA-*oPaFADS17*

**Figure 6:**
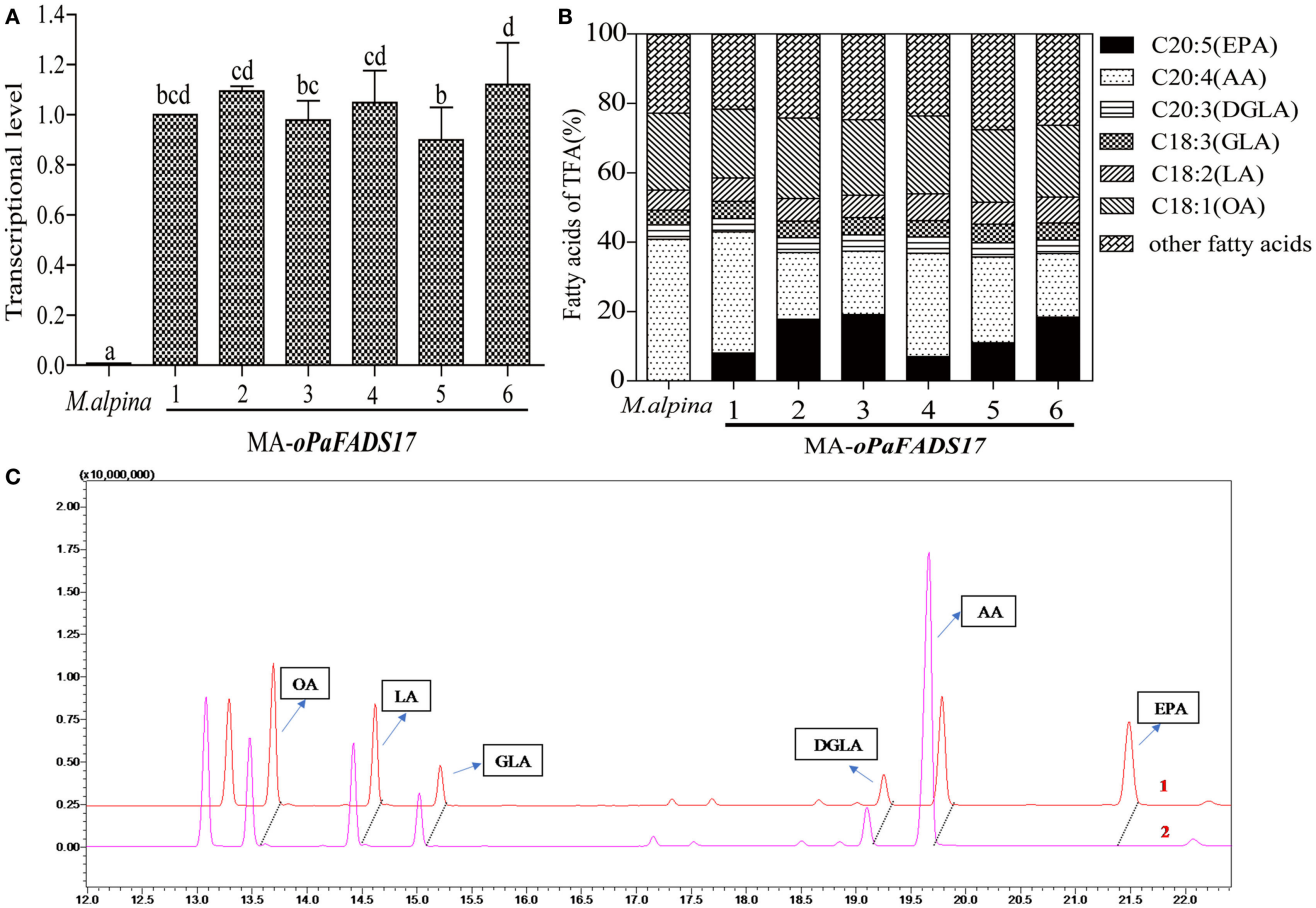
Relative expression levels of *oPaFADS17* gene and fatty acid production and composition in *Mortierella alpina* transformants. **(A)** Relative expression levels; **(B)** fatty acid production and composition; and **(C)** gas chromatographs of the transformant (1) and wild-type *M. alpina* (2). All strains were cultivated in 100 mL of liquid broth medium for 7 days at 28°C.

RT-qPCR analysis of *oPaFADS17* transcript levels revealed the successful transcription of this construct in all heterologous expression strains, whereas no transcription was detected in wild-type *M. alpina* (Figure 6A). The stable transformant and wild-type *M. alpina* strains were cultivated in broth medium for 7 days at 28°C prior to the evaluation of fatty acid productivity and composition. These strains had similar fatty acid components but significantly different AA and EPA contents (Figures 6B,C), and the latter differed among transformants according to differences in integration sites. High EPA-producing transformants exhibited sharp decreases in AA and dramatic increases in EPA level. Among all transformants, MA-*oPaFADS17-3* (CCFM 695) produced yields of 18.7% EPA and 18.8% AA among TFA (Table 3), indicating the conversion of almost half of the AA present in *M. alpina* and an EPA production of 617 mg L^−1^.

**Table 3:** Effect of culture medium on biomass, total lipid, and EPA production by *M. alpina* CCFM 695.

Groups			Dry biomass (g L^**−**1^)	TFA (g L^**−**1^)	EPA (%)	EPA (mg L^**−**1^)	AA (%)	AA (mg L^**−**1^)
Carbon source	Glucose	50 g L^−1^	10.90 ± 0.10^b^	3.30 ± 0.06^c^	18.71 ± 0.20^a^	617.12 ± 14.58^a^	18.80 ± 0.72^d^	619.97 ± 45.24^c^
	Corn starch	50 g L^−1^	12.53 ± 0.35^c^	5.39 ± 0.01^d^	22.96 ± 1.42^ab^	1237.61 ± 76.68^c^	13.89 ± 1.17^c^	748.67 ± 63.24^d^
	soluble starch	50 g L^−1^	14.50 ± 0.10^d^	2.80 ± 0.05^b^	33.44 ± 1.15^c^	935.71 ± 18.41^b^	7.03 ± 0.76^a^	196.92 ± 24.11^a^
	potato starch	50 g L^−1^	7.33 ± 0.32^a^	2.13 ± 0.02^a^	32.47 ± 2.35^c^	692.78 ± 42.75^a^	10.44 ± 1.16^b^	222.91 ± 27.16^a^
	Glycerol	50 g L^−1^	8.10 ± 0.28^a^	3.39 ± 0.15^c^	17.63 ± 0.60^a^	604.27 ± 4.53^a^	14.50 ± 0.31^c^	497.54 ± 23.95^b^

Nitrogen source	DSOM	10 g L^−1^	5.15 ± 0.35^a^	2.58 ± 0.08^a^	18.69 ± 0.24^a^	481.96 ± 18.82^a^	12.06 ± 0.14^a^	310.51 ± 17.73^a^
		30 g L^−1^	14.25 ± 0.92^b^	6.85 ± 0.19^b^	19.55 ± 0.9^a^	1339.25 ± 70.75^c^	12.32 ± 1.03^a^	842.59 ± 46.79^b^
		50 g L^−1^	14.20 ± 0.42^b^	6.56 ± 0.14^b^	23.27 ± 0.55^bc^	1551.82 ± 34.64^d^	15.04 ± 0.14^b^	980.07 ± 20.95^c^
		70 g L^−1^	13.70 ± 0.14^b^	5.23 ± 0.12^b^	20.67 ± 0.72^ab^	1089.7 ± 39.83^b^	16.13 ± 0.24^b^	840.46 ± 34.95^b^

	KNO_3_	0 g L^−1^	9.93 ± 0.57^ab^	4.94 ± 0.33^ab^	22.14 ± 1.15^b^	1093.45 ± 70.10^b^	15.26 ± 0.78^a^	753.60 ± 41.66^a^
		5 g L^−1^	10.60 ± 0.62^ab^	4.88 ± 0.55^ab^	18.75 ± 0.89^a^	912.18 ± 59.66^a^	16.21 ± 0.29^ab^	791.56 ± 102.92^ab^
		10 g L^−1^	9.47 ± 0.32^a^	4.50 ± 0.35^a^	19.42 ± 0.49^a^	873.78 ± 84.49^a^	17.09 ± 0.37^b^	768.54 ± 68.44^a^

*Culture conditions: pH: 6.0; temperature: 28°C; cultivation period: 7 days; DSOM, defatted soybean meal, used as organic nitrogen*.

*Values are mean ± SD, n = 3. Values in the same column that do not share the same alphabetic superscripts are significantly different at 5% levels according to Tukey’s test*.

### Effects of Carbon Sources to EPA Production

Broth medium (pH 6.0) was used in studies of the effects of different carbon sources on biomass, lipid, and EPA production by *M. alpina* CCFM 695. Of the tested sources, soluble starch yielded the highest level of mycelial growth (14.50 g L^−1^; Table 3), whereas maximum EPA production was achieved in medium containing corn starch (1.2 g L^−1^). Starch, an energy storage polysaccharide produced widely by plants such as wheat, maize, rice, and potato, is one of the most abundant carbohydrates in nature (Ledesma-Amaro and Nicaud, 2016). As reported previously, glucose was the optimal carbon source for fatty acid accumulation by *M. alpina*, whereas starch was a poor growth supporter and only allowed the generation of 1.6 g L^−1^ dry biomass (Nisha and Venkateswaran, 2011). In contrast, our results showed that a medium containing soluble starch or corn starch generated much higher biomass levels relative to glucose (10.9 g L^−1^; Table 3), which we attribute to potential differences among strains. However, soluble starch was not conducive to TFA production (2.8 g L^−1^; Table 3). Potato starch yielded the lowest amount of dry cell biomass, and glycerol yielded the poorest EPA production by *M. alpina* CCFM 695. In summary, various carbon sources had specific effects on EPA production.

### Effects of Nitrogen Sources to EPA Production

Broth medium was also used to study the effects of nitrogen sources on biomass, lipid, and EPA production by *M. alpina* CCFM 695. In these experiments, yeast extract was replaced by different concentrations of DSOM (Table 3), an abundant and inexpensive agricultural product. However, as DSOM contains insoluble materials and the lipids are intracellular products of fungal cells, the direct addition of soy flour to the medium would increase the difficulty of biomass separation from soybean solids. We therefore supplemented the culture media with a filtrate of DSOM that had been boiled for 20 min. The addition of 50 g L^−1^ of DSOM promoted *M. alpina* CCFM 695 growth and led to an increase in biomass to 14.2 g L^−1^, a much greater elevation than that observed with yeast extract. Furthermore, DSOM increased EPA production by more than twice over yeast extract (1.5 g L^−1^ vs. 617 mg L^−1^). In other words, DSOM might not only decrease the cost of fermentation, but it could also serve as a good nitrogen source for *M. alpina* CCFM 695 in the industrial production of EPA. Furthermore, a study of the inorganic nitrogen KNO_3_ concentration revealed that a low concentration of this component could promote EPA accumulation.

### Range Analysis

Nine experiments were conducted per the OA_9_ matrix, and the resulting of dry biomass, TFA and EPA production were presented in Table 4. EPA production varied from 376 to 1,734 mg L^−1^, dry biomass and TFA content increased to 16.55 and 6.46 g L^−1^, respectively. Table 5 listed the mean values of *K* (k*_ji_*) for different factors at different levels in the range analysis. A higher mean k*_ji_* value indicated that the corresponding level had a greater effect on EPA production. Therefore, we determined the following optimal parameters: the optimal carbon source was glucose, the optimal DSOM concentration was 50 g L^−1^, and the optimal KNO_3_ concentration was 5 g L^−1^ (A_1_B_2_C_2_). This combination yielded EPA production level as high as 1.73 g L^−1^. Meanwhile, the *R_j_* value demonstrates the significance of a factor’s influence, and a larger value indicates that the factor had a greater effect on EPA production. In Table 5, the factor significance level, in decreasing order, was: Carbon source (*R*_A_) > DSOM concentration (*R*_B_) > KNO_3_ concentration (*R*_C_). The range value *R*_A_ was the largest, indicating that the carbon source had the most significant effect on EPA production.

**Table 4:** Results of OA_9_ matrix orthogonal test.

Trial no.	Factors			Dry biomass (g L^**−**1^)	TFA (g L^**−**1^)	EPA (g L^**−**1^)
	A	B	C			
1	3	2	3	5.85	2.455	0.603
2	3	3	1	8.90	3.473	0.911
3	2	1	3	9.20	3.563	0.939
4	2	3	2	11.80	3.696	1.275
5	2	2	1	10.60	4.063	1.001
6	1	3	3	13.75	5.230	1.090
7	1	1	1	7.40	3.235	0.761
8	3	1	2	4.15	1.711	0.376
9	1	2	2	16.55	6.460	1.734

**Table 5:** Range analysis data.

Target (g L^**−**1^)	Value name	Factors		
		A	B	C
EPA	*K*_1_	3.585	2.076	2.673
	*K*_2_	3.215	3.338	3.385
	*K*_3_	1.890	3.276	2.632
	*k*_1_(*K*_1_/3)	1.195	0.692	0.891
	*k*_2_(*K*_2_/3)	1.072	1.113	1.128
	*k*_3_(*K*_3_/3)	0.630	1.092	0.877
	*R_j_*	0.565	0.421	0.251
	Optimal scheme	A_1_	B_2_	C_2_

## Discussion

The oleaginous fungus *M. alpina* may contain an AA fraction as high as 50% of the TFA. Accordingly, researchers have attempted to enhance EPA production in this species by overexpressing endogenous ω-3 fatty acid desaturase (Okuda et al., 2015). Despite an abundance of endogenous ω-3 fatty acid desaturase substrate, however, this strain produces little EPA even at low temperatures, possibly consequent to a low ω-3 fatty acid desaturase expression level and/or a low AA conversion efficiency. The *M. alpina* PUFAs synthesis pathway (Figure 1) exhibits a strong preference for LA of FADS6 and low Δ-15 activity from ω-3 desaturase. As a result, LA flows easily to the next step, allowing a large accumulation of AA and providing sufficient substrate for EPA synthesis by a ω-3 desaturase with high Δ-17 activity. Although EPA could be produced *via* the ω-6 pathway, further accumulation could be achieved through the ω-3 pathway, a process that was attempted in our lab *via* the heterogeneous expression of ALA-preferring FADS6 and exogenous ALA supplementation. Finally, an EPA production of 588.5 mg L^−1^ was achieved (Shi et al., 2016). In this study, we transformed a Δ-17 fatty acid desaturase PaD17 into *M. alpina*, and achieved a AA-to-EPA conversion rate as high as 49.8% at room temperature, with EPA fractions comprising nearly 20% of TFA. In the future, the coexpression of multiple heterologous genes related to PUFAs biosynthesis, such as FADS6 and ω-3 fatty acid desaturase with high Δ-15 and Δ-17 activities, could lead to much larger EPA yields. Additionally, a high EPA content could be obtained *via* metabolic engineering by applying high-expression promoters and blocking undesired fatty acid synthesis.

Compared with our previous study (Huang et al., 2014), we observed different effects of heterologous ω-3 fatty acid desaturase PaD17 and endogenous ω-3 fatty acid desaturase FADS15 on EPA accumulation, which were probably attributable to structural diversity. Currently, the membrane structures of ω-3 fatty acid desaturases remain unknown because of technical difficulties associated with purification. A better understanding of structural-functional relationships would not only introduce further possibilities regarding the manipulation of fatty acid enzyme activities and substrate specificities, but could also lead to the commercial production of novel, highly valuable, and industrially important fatty acids. This research would also pave the way for the generation of transgenic livestock that express essential ω-3 LC-PUFAs.

Our ability to generate high levels of EPA at room temperature, rather than under low temperature conditions, indicates the potential for greatly reducing production costs and suitability for industrial EPA production. Moreover, the use of inexpensive DSOM, an agricultural waste by-product, as an organic nitrogen source for *M. alpina* CCFM 695 culture, further decreased the fermentation costs. Consequently, EPA production increased to 1.7 g L^−1^ at room temperature in medium supplemented by inexpensive DSOM, thus a single gram of DSOM was converted to 34 mg of high-value EPA. The method described in this article may therefore be an economical and sustainable strategy to satisfy future dietary needs.

## Ethics Statement

This article does not contain any studies with human participants or animals performed by any of the authors.

## Author Contributions

CG carried out the experiments and drafted the manuscript. HC, TM, XT, LC, and ZG performed the complementation experiments. HC and YC analyzed the data and helped to draft the manuscript. WC and HZ conceived and coordinated the study and revised the manuscript. All authors read and approved the final manuscript.

## Conflict of Interest Statement

The authors declare that the research was conducted in the absence of any commercial or financial relationships that could be construed as a potential conflict of interest.

## Abbreviations

LC-PUFAs, long-chain polyunsaturated fatty acids; LA (C18:2, ω-6), linoleic acid; GLA (C18:3, ω-6), γ-linolenic acid; DGLA (C20:3, ω-6), dihomo-γ-linolenic acid; AA (C20:4, ω-6), arachidonic acid; ALA (C18:3, ω-3), α-linolenic acid; SDA (C18:4, ω-3), stearidonic acid; ETA (C20:4, ω-3), eicosateteraenoic acid; EPA (C20:5, ω-3), eicosapentaenoic acid; DHA (C22:5, ω-3), docosahexaenoic acid; TFA, total fatty acids; FAME, Fatty acid methyl ester; DSOM, defatted soybean meal.
